# The efficacy and tolerability of rotigotine on patients with periodic limb movement in sleep: A systematic review and meta-analysis

**DOI:** 10.1371/journal.pone.0195473

**Published:** 2018-04-18

**Authors:** Meng-Ni Wu, Ping-Tao Tseng, Tien-Yu Chen, Yen-Wen Chen, Li-Min Liou, Pao-Yen Lin, Chung-Yao Hsu

**Affiliations:** 1 Department of Neurology, Kaohsiung Medical University Hospital, Kaohsiung, Taiwan; 2 Department of Master’s Program in Neurology, Faculty of Medicine, Kaohsiung Medical University, Kaohsiung, Taiwan; 3 Department of Psychiatry, Tsyr-Huey Mental Hospital, Kaohsiung, Taiwan; 4 WinShine Clinics in Specialty of Psychiatry, Kaohsiung City, Taiwan; 5 Department of Psychiatry, Tri-Service General Hospital, School of Medicine, National Defense Medical Center, Taipei, Taiwan; 6 Prospect Clinic for Otorhinolaryngology & Neurology, Kaohsiung, Taiwan; 7 Department of Psychiatry, Kaohsiung Chang Gung Memorial Hospital and Chang Gung University College of Medicine, Kaohsiung, Taiwan; 8 Center for Translational Research in Biomedical Sciences, Kaohsiung Chang Gung Memorial Hospital, Kaohsiung, Taiwan; University of Rome Tor Vergata, ITALY

## Abstract

**Objective:**

There is still no consensus on the treatment for periodic limb movement in sleep (PLMS). This study aimed to determine the efficacy and tolerability of rotigotine in patients suffering from PLMS.

**Methods:**

Publications listed in PubMed, ScienceDirect, The Cochrane Library, and ClinicalTrials.gov were reviewed to assess the efficacy of rotigotine on PLMS. Analyses of PLMS frequency before and after rotigotine treatments (pre- and post-intervention studies) and PLMS frequency between placebo and rotigotine treatments (placebo-controlled trial studies) were included in our study. A systematic review and meta-analysis was conducted.

**Results:**

Five publications involving 197 participants were included in this study. Among these articles, pre- and post-intervention data involving 55 participants were available from three articles, while placebo-controlled trial data from 107 participants receiving rotigotine and 70 participants receiving a placebo were available from an additional three articles. In the pre- and post-intervention studies, the periodic limb movement index was significantly decreased after therapy with rotigotine with a difference in means of −5.866/h (95% CI, −10.570 to −1.162, *p* = 0.015). In comparison with the placebo, the use of rotigotine significantly lowered the periodic limb movement index, with a difference in means of −32.105/h (95% CI, −42.539 to −21.671, *p* < 0.001), reduced the PLMS with arousal index, with a difference in means of −7.160/h (95% CI, −9.310 to −5.010, *p* < 0.001), and increased the withdrawal rate, with an odds ratio of 3.421 (95% CI, 1.230 to 9.512, *p* = 0.018).

**Conclusions:**

This meta-analysis revealed the considerable efficacy of rotigotine in alleviating the frequency of PLMS. However, the high withdrawal rate should be taken into account.

## Introduction

Periodic limb movement in sleep (PLMS), which is characterized by episodes of repetitive and stereotypical leg movements involving rhythmic extension of the big toe and dorsiflexion of the ankle with occasional concomitant knee and hip flexion, is common in the general population, with a prevalence of 5–11% [1]. Only a portion of people showing PLMS on polysomnography experienced clinical symptoms, including disturbed sleep quality or excessive daytime sleepiness. Clinical symptoms exclusively resulting from PLMS are defined as periodic limb movement disorder (PLMD), which has a prevalence of 3.9% [1, 2]. PLMS can be recorded alone or concomitantly recorded in 27.6% of patients with obstructive sleep apnea (OSA), 70% of patients with rapid eye movement sleep behavior disorder (RBD), and 80.2% of patients with restless legs syndrome (RLS)[3–6]. Clinically, it is difficult to distinguish whether symptoms are resulted from PLMS or concurrent sleep disorders. However, to date, it is reported that pharmacological therapy is only indicated in patients with PLMD [7].

There is growing evidence implying the clinical importance of PLMS. The presence of PLMS was reported to be associated with impaired quality of life, insomnia, and depressive symptoms in patients with RLS [8, 9]. In patients with RBD, the presence of PLMS was reported to be related to disrupted sleep architecture without aggravation of daytime sleepiness [10]. Additionally, the presence of PLMS, either in combination with concurrent sleep disorders or not, has been reported to be associated with hypertension [11], congestive heart failure [12, 13], coronary and peripheral artery diseases [13–15], and increased levels of inflammatory factors, including C-reactive protein [16], fibrinogen [16], and lipoprotein-associated phospholipase A2[17]. In patients with congestive heart failure and chronic kidney diseases, the presence of PLMS independently predicted mortality [18, 19]. Furthermore, the frequency of PLMS, which is quantified by the periodic limb movement index (PLMI), was significantly correlated with the prevalence of hypertension [20], Framingham cardiovascular risk [21], and levels of inflammatory factors [16, 17] and was a predictor of myocardial infarction [15]. Therefore, it seems unreasonable to overlook PLMS, either with or without concurrent sleep disorders.

Considering the high prevalence of PLMS in patients with RLS and the association of genetic risk factors between PLMS and RLS [22], it is hypothesized that RLS and PLMS may share a similar pathophysiology in which dopaminergic dysfunction in A11 area was involved followed by disinhibition of serotonergic dorsal raphe descending neurons and the intermediate lateral cell column [23]. Impairment of central dopaminergic transmission in PLMS can also explain the co-occurrence of PLMS in patients with idiopathic RBD, whose neuroimaging studies reveal decreased striatal dopaminergic innervation [6].

On the basis of a possible mechanism of central dopaminergic dysfunction in patients with PLMS, the efficacy of dopaminergic receptor agonists and levodopa on the PLMI have been widely investigated [24–31]. The D2-like dopaminergic receptors, including D2, D3, and D4 receptors, play an important role in PLMS, and the efficacy of dopaminergic receptor agonists targeting the D3 receptor on PLMS is better than that achieved when D2 receptors are targeted [23, 32]. Therefore, recent studies investigating PLMS therapies have focused on non-ergot dopaminergic receptor agonists that specifically target the D3 receptor [33–35]. Clinical trials have reported that non-ergot dopaminergic receptor agonists, including pramipexole [24–26], ropinirole [27–29], and rotigotine [30] significantly decreased the PLMI. Meta-analyses have reported that pramipexole and ropinirole can reduce the PLMI in patients with RLS [36, 37]. However, no meta-analysis, to the best of our knowledge, aimed to assess the efficacy of rotigotine in reducing PLMI [30, 36, 37]. Unlike pramipexole and ropinirole, which must be taken orally, rotigotine can be administered through transdermal patches that successfully maintain stable plasma levels over the course of a day in a manner that physicians perceive to be advantageous [38, 39]. Thus far, rotigotine has not been comprehensively reviewed to explore its role in the alleviation of PLMS. Therefore, we conducted a systematic review and meta-analysis to determine the efficacy and tolerability of rotigotine in patients afflicted by PLMS.

## Materials and methods

The meta-analysis was conducted according to the guidelines presented in the *Preferred Reporting Items for Systematic Reviews and Meta-Analyses* (PRISMA) statement[40] (data in S1 Table). This study was approved by Institutional Review Board of the Tri-Service General Hospital (TSGHIRB: B-105-12). On the basis of nature characteristics of meta-analysis, the informed consent is not applicable.

### Database searches and identification of eligible papers

Two independent authors (MN Wu and PT Tseng) searched PubMed, ScienceDirect, The Cochrane Library, and ClinicalTrials.gov using the keywords “(PLMS OR periodic limb movement syndrome) AND (Rotigotine)” for articles published before July 19, 2017. Furthermore, the reference lists of review articles or clinical guidelines relevant to this topic were hand searched to identify potentially eligible papers [41–47].

Two independent authors (MN Wu and PT Tseng) reviewed titles and abstracts for eligibility. A list of potentially eligible studies was constructed by consensus, after which full text examinations were conducted. A third reviewer (CY Hsu) was consulted if any inconsistencies arose.

### Inclusion and exclusion criteria

The inclusion criteria applied to this study were as follows: (1) published articles investigating the efficacy of rotigotine on PLMS, either in the form of a pre- and post-intervention comparison design or placebo-controlled trial studies, and (2) articles that reported on clinical trials conducted in humans. We did not set any limitations.

We excluded animal studies.

### Methodological quality appraisal

The methodological quality of the included studies was evaluated using tools of the Jadad scale[48]. The Jadad scores were calculated for each study and consist of three-categories of study quality, including randomization, blinding, and withdrawals. The Jadad score ranges from zero (poor quality) to five (high quality).

### Primary outcomes

The primary outcome measure was the change in frequency of PLMS, which was defined as the rate of PLMI (PLM/h) as recorded using polysomnography.

### Secondary outcomes

Secondary outcomes of interest included changes in sleep parameters, such as PLMAI, percentage of time spent in the deep sleep phase, percentage of time spent in the REM sleep phase, Epworth Sleepiness Scale, sleep efficiency, and sleep onset latency.

### Data extraction and management

Two independent authors extracted data from the eligible studies into a database of pre-determined variables of interest. The variables extracted included mean age (years), gender, body mass index (BMI), mean rotigotine dosage (mg/day), rotigotine treatment duration (week), ethnicity (Caucasian, Africa American, Hispanic, Asian, or Native America), and Jadad scores.

When data were not found in the articles, we tried to contact the corresponding authors electronically to request additional data on at least two different occasions.

### Meta-analysis

Based on the presumed heterogeneity among the included studies, data were analyzed using random-effects meta-analysis models rather than fixed effects models [49] on the Comprehensive Meta-Analysis software version 3 platform (Biostat, Englewood, NJ). Because all the effect sizes (ESs) of the primary outcomes were measured on the same scale (PLMI), we analyzed the ESs of the changes in PLMI both before and after rotigotine treatment and the differences between rotigotine and placebo using differences in means and 95% confidence intervals (95% CIs) rather than Hedges’ *g*, because the former was directly measured by clinicians. We calculated the odds ratio (OR) with 95% CIs for secondary outcomes using dichotomous items (such as the drop-out rate). We investigated the ESs of both the changes in PLMAI before and after rotigotine treatment and the differences between rotigotine and placebo using difference in means and 95% CIs.

We considered the statistical result to be “statistically significant” when the two-tailed *p* values were less than 0.05. Heterogeneity was evaluated using the Q statistic[50]. We examined publication bias by visual inspection of funnel plots [51] and Egger’s regression tests [52]. We performed the Duval and Tweedie trim and fill test to adjust ESs when evidence of publication bias was found [53].

To evaluate the potential sources of heterogeneity and confounding effects, we performed meta-regression and subgroup meta-analyses. Specifically, when there were at least five datasets, we conducted the meta-regression procedure using the unrestricted maximum likelihood method. The variables of interest for meta-regression were mean age (years), gender, BMI, mean rotigotine dosage (mg/day), rotigotine treatment duration (week), ethnicity (Caucasian, African American, Hispanic, Asian, or Native America), and Jadad scores. All of the meta-analyses or subgroup meta-analyses were performed under on at least three datasets [54].

We also measured the sensitivity of analyses using the one study removal test, which has been widely used in meta-analysis. More specifically, we excluded one study at a time from the analyses to determine whether an outlier could be biasing our ES estimates [55].

## Results

### Study selection

**Fig 1:**
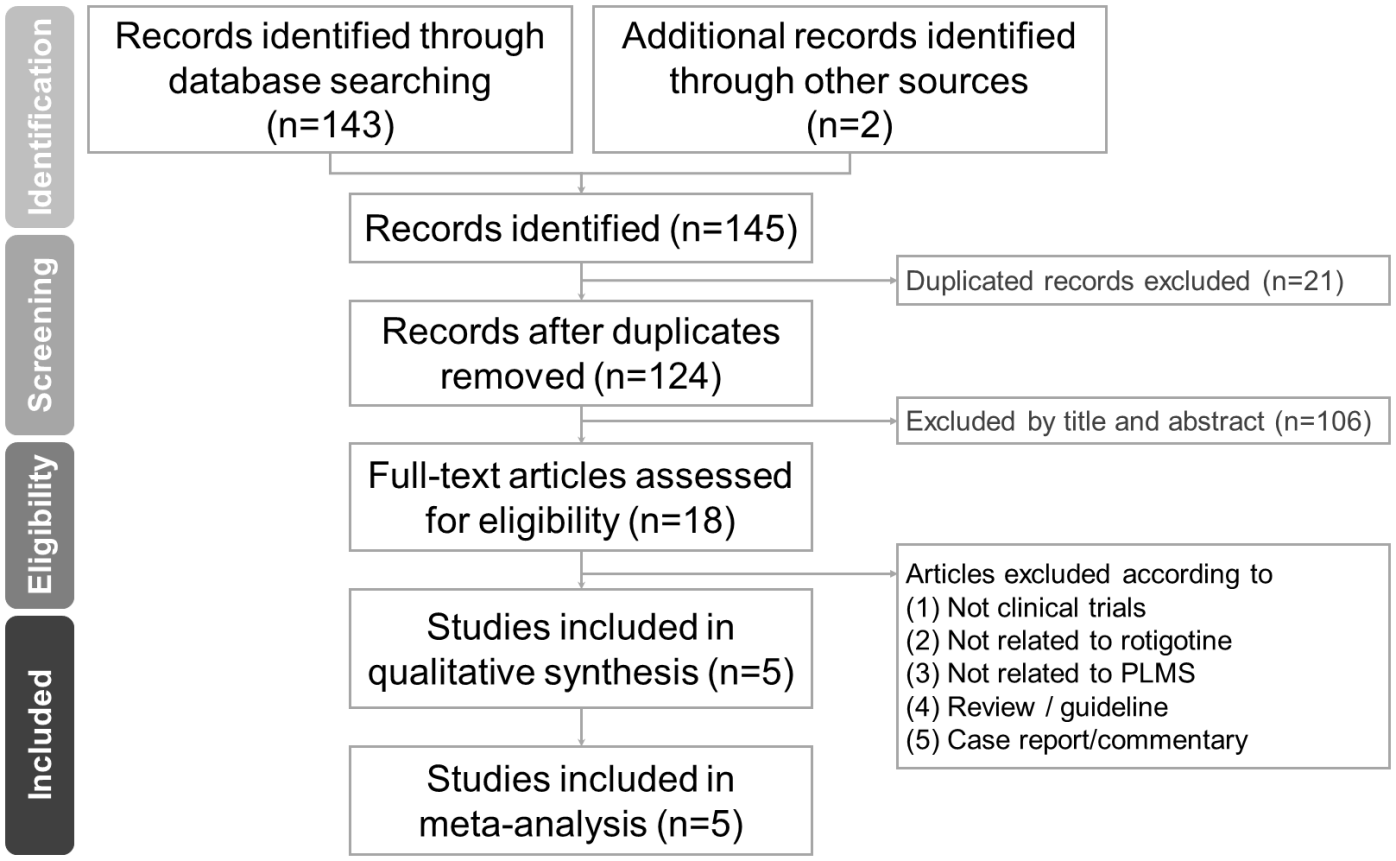
Flowchart of the selection strategy for meta-analysis according to PRISMA guidelines.

The PRISMA flowchart used for study selection in this systematic review is shown in Fig 1. After exclusion of duplicates, 18 full text articles were assessed for eligibility. Among them, thirteen were excluded because the eligibility criteria were not met (S2 Table). Therefore, five articles were eligible for the current meta-analysis (Table 1) [30, 56–59].

**Table 1:** Summary of characteristics of the studies included in the meta-analysis.

Study	Diagnosis	Criteria of PLMS	Comparison	N	% female	Mean age	Dose (mg/day)	Duration of rotigotine (weeks)	Country
Wang. Y (2016)	PD + RBD	N/A	Pre-post treatment	11	27.3	66.3 +/− 8.5	12.4 ± 4.3	24.7 ± 2.4	China
Elshoff, J.P (2016)	Idiopathic RLS	> or = 5	Pre-post treatment	23	62.5	15.3 +/− 1.3	Gradually titrate*	23/24 complete 4 weeks trials	United States
Dauvilliers, Y (2016)	ESRD + RLS	> or = 15	Pre-post treatment Rotigotine vs placebo	20 10	35.0 30.0	57.2 +/− 12.6 50.7 +/− 16.3	2.31	25/30 complete 10-week trials	Multiple countries
Oertel, W.H (2010)	Idiopathic RLS	> or = 15	Pre-post treatment Rotigotine vs placebo	46 20	76.0 70.0	60.8 +/− 9.4 56.3 +/− 9.8	2.1 ± 0.8	7.0 ± 1.6	Multiple countries
Bauer, A (2016)	RLS	> or = 15	Rotigotine vs placebo	41 40	62.5 65.0	57.8 +/− 11.2 57.4 +/- 9.1	2.5	7.4 ± 1.2	Germany

*: Patients received rotigotine in dosage of 0.5 mg/24 h in week 1 (2.5-cm2 patch), 1 mg/24 h in week 2 (5-cm2 patch), 2 mg/24 h in week 3 (10-cm2 patch), and 3 mg/24 h in week 4 (15-cm2 patch)

Data presentation: mean +/− SD

Abbreviations: SD: Standard deviation; N/A: Not available; RBD: REM sleep behavior disorder; RLS: Restless legs syndrome; ESRD: end-stage renal disease; PD: Parkinson’s disease; PLMI: periodic limb movement index; PLMS: periodic limb movement during sleep; PLC: placebo

Among the five eligible articles, three presented data detailing the changes in PLMI before and after rotigotine treatment (pre- and post-intervention studies; participants = 55, mean age = 40.7, mean female proportion = 45.5%) [56–58]. Three articles were eligible for the meta-analysis of placebo-controlled rotigotine treatment effects (placebo-controlled trial studies; rotigotine participants = 107, mean age = 59.0, mean female proportion = 63.2%; placebo participants = 70, mean age = 56.1, mean female proportion = 61.4%) [30, 58, 59].

The duration of rotigotine titration was 3 weeks in three studies, 4 weeks in one study, and 8 weeks in another study. The duration of rotigotine maintenance was 4 weeks in two studies, 12–20 weeks in one study, and 2 weeks in one study, while one study did not present any data on this metric. The maximal dosage of rotigotine was 3 mg/24 h in four studies and 16 mg/24 h in one study.

### Methodological quality of included studies

Among the five studies, the average Jadad scores were 2.6 with a standard deviation (SD) of 1.7 (S3 Table).

### Meta-analysis of rotigotine effects on PLMI

#### Primary outcome: PLMI

**Fig 2:**
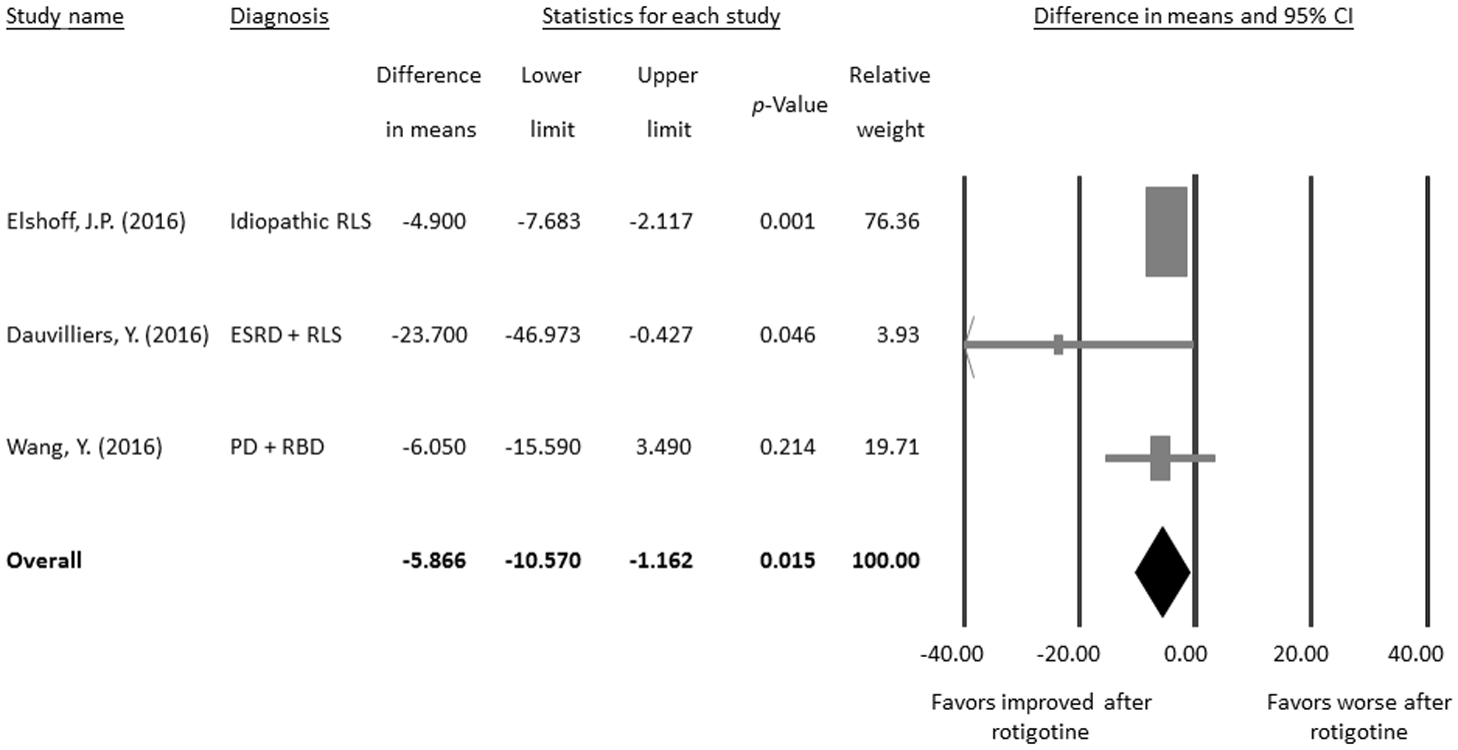
Meta-analysis of changes in PLMI after rotigotine treatment.

When we focused on the three eligible pre- and post-intervention studies [56–58], the meta-analysis found significantly decreased PLMI scores after rotigotine treatment in patients with PLMS (difference in means = −5.866, 95% CI = −10.570 to −1.162, *p* = 0.015; Fig 2) without significant heterogeneity (Q value = 2.502, df = 2, *p* = 0.286; *I*^*2*^ = 20.058%, tau = 2.351) or publication bias via Egger’s regression (t value = 1.708, df = 1, *p* = 0.337) or funnel plot (S1A Fig).

#### Sensitivity test

The sensitivity test by means of the one study removal method revealed that the main results of the meta-analysis were not significant after removal of the Elshoff, (2016) (difference in means = −11.552, 95% CI = −27.575 to 4.472, *p* = 0.158) [57] or Wang, (2016) studies (difference in means = −10.604, 95% CI = −27.543 to 6.336, *p* = 0.220) [56], which might be due to the smaller sample size that results from removal of any study from this meta-analysis.

#### Meta-regression

The meta-regression procedure could not be performed because there were fewer than five datasets.

### Meta-analysis of rotigotine effects on PLMI compared to placebo

#### Primary outcome: PLMI

**Fig 3:**
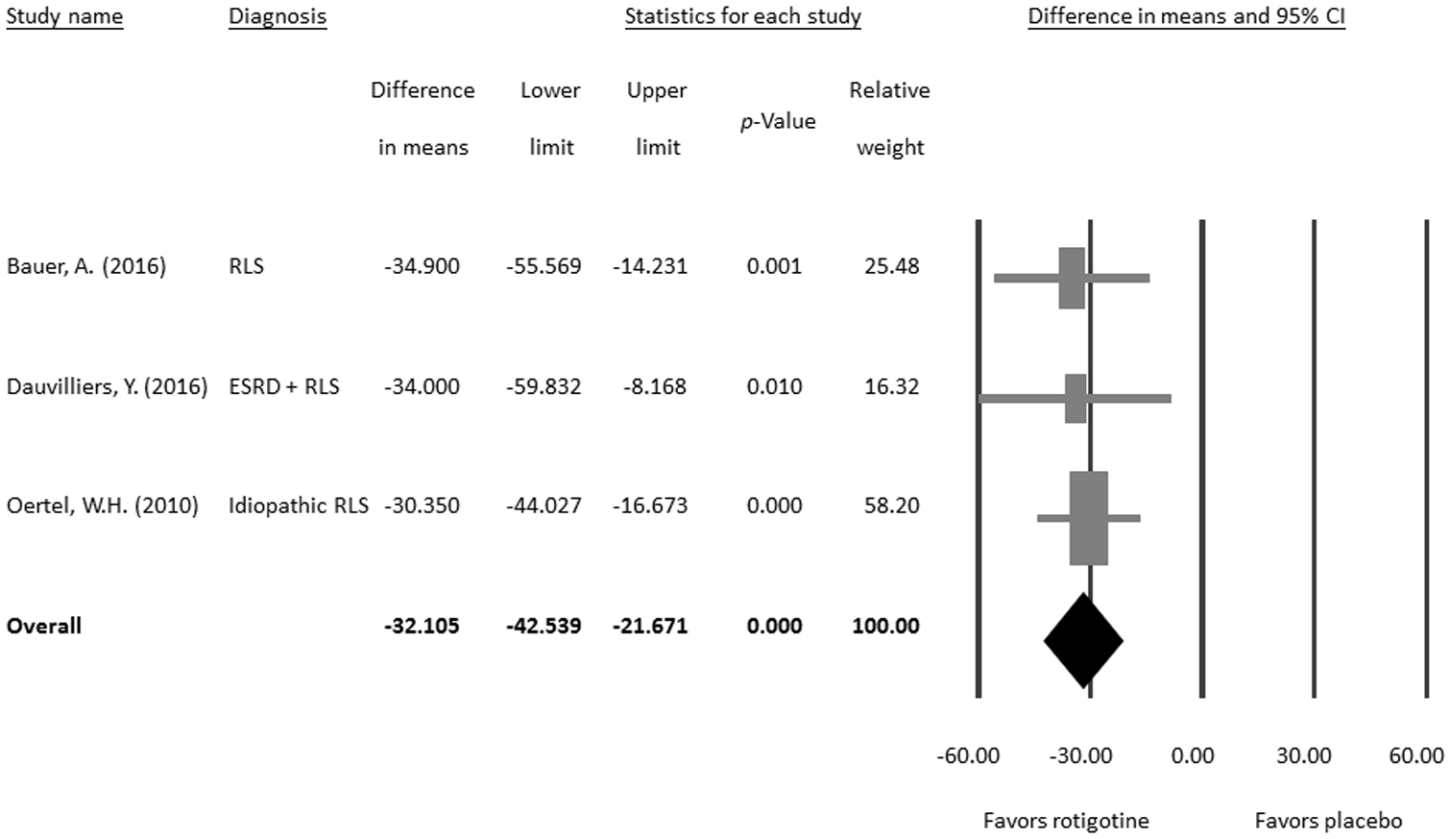
Meta-analysis of differences in PLMI between rotigotine and placebo treatments.

When taking into account the three eligible placebo-controlled trial studies [30, 58, 59], the meta-analysis revealed that the treatment effect of rotigotine on PLMI scores is significantly better than that of placebo (difference in means = −32.105, 95% CI = −42.539 to −21.671, *p* < 0.001; Fig 3) without significant heterogeneity (Q value = 0.154, df = 2, *p* = 0.926; *I*^*2*^ < 0.01%, tau <0.01) or publication bias via Egger’s regression (t value = 1.886, df = 1, *p* = 0.310) or funnel plot (S1B Fig).

#### Secondary outcome: PLMAI

**Fig 4:**
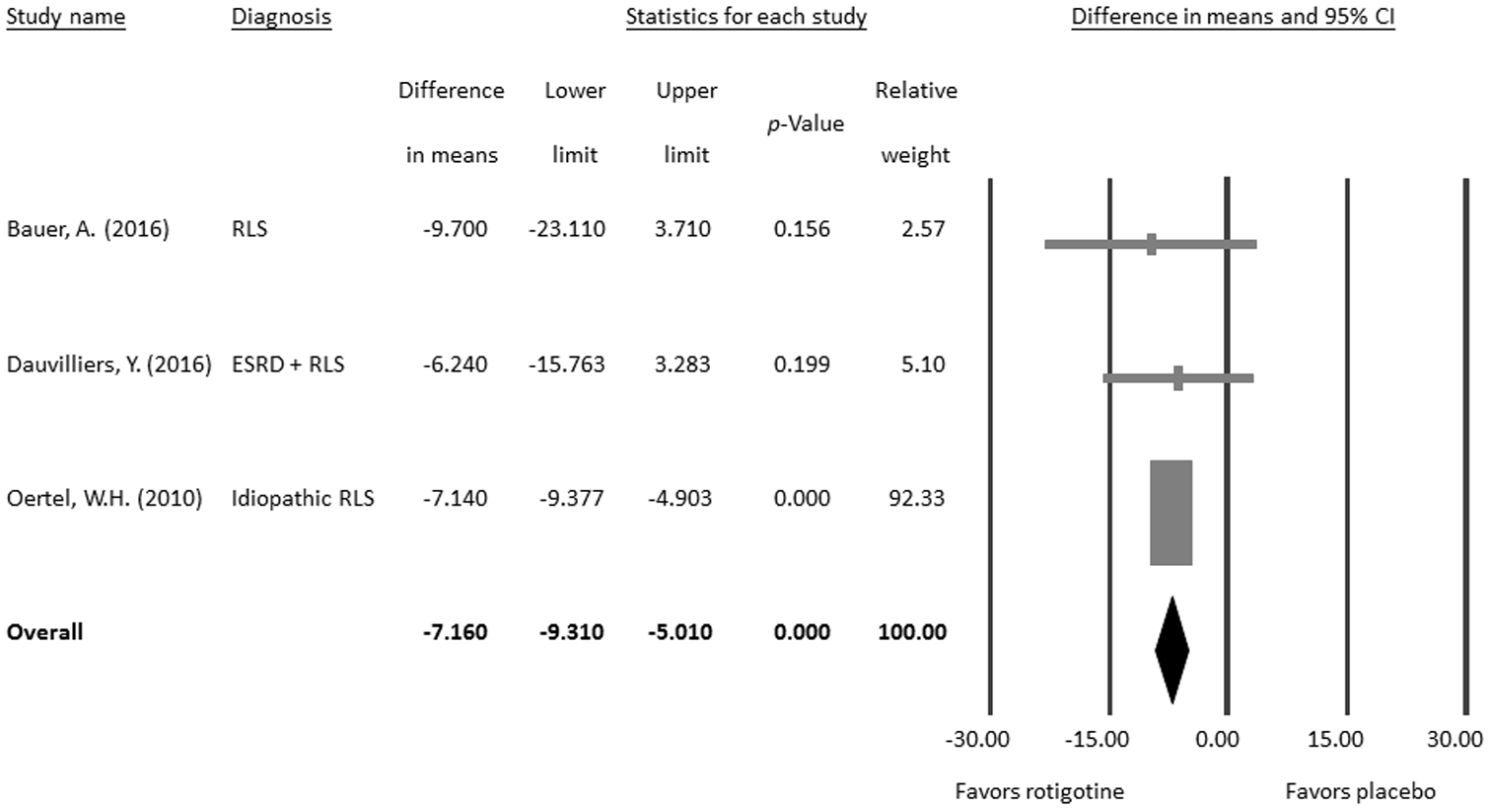
Meta-analysis of differences in PLMAI between rotigotine and placebo-treated groups.

The subgroup meta-analysis of the PLMAI results revealed that the treatment effects of rotigotine on the PLMAI are significantly different from that of placebo controls (difference in means = −7.160, 95% CI = −9.310 to −5.010, *p* < 0.001; Fig 4) without significant heterogeneity (Q value = 0.174, df = 2, *p* = 0.917; *I*^*2*^ < 0.01%, tau <0.01) or publication bias via Egger’s regression (t value = 0.388, df = 1, *p* = 0.764) or funnel plot (S1C Fig).

#### Secondary outcome: Withdrawal rate

**Fig 5:**
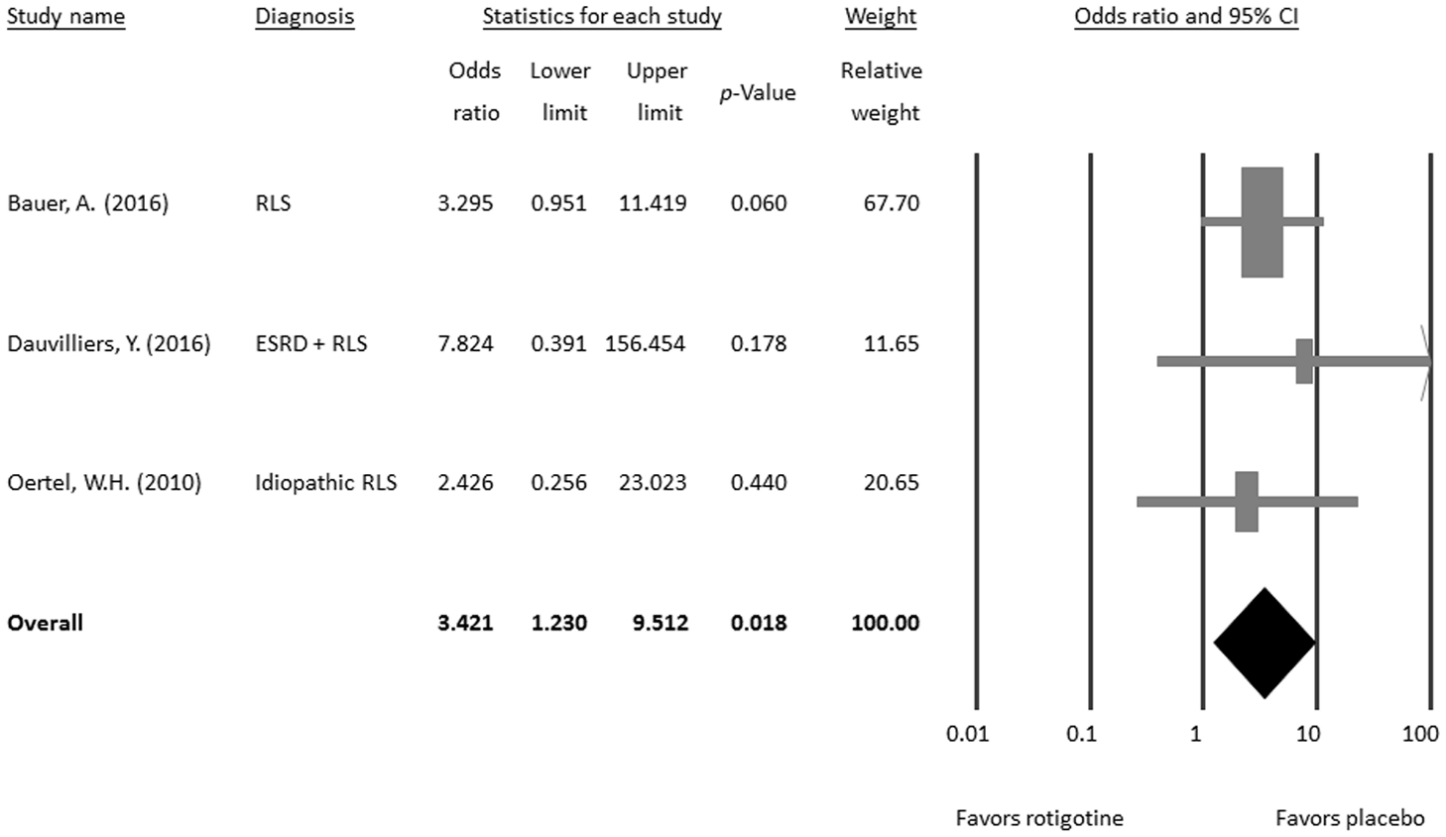
Meta-analysis of the withdrawal rate of the rotigotine and placebo-treated groups.

Subgroup meta-analysis showed that the withdrawal rate was significantly higher in the rotigotine-treated group than in the placebo controls (OR = 3.421, 95% CI = 1.230–9.512, *p* = 0.018; Fig 5) without significant heterogeneity (Q value = 0.386, df = 2, *p* = 0.824; *I*^*2*^ < 0.01%, tau < 0.01) or publication bias via Egger’s regression (t value = 0.543, df = 1, *p* = 0.684) or funnel plot (S1D Fig).

#### Adverse events

In the pre- and post-intervention treatment studies, the rate of adverse effects was 27.3% and 45.8%, whereas in the placebo-controlled trial studies, the rate of adverse effects was 60%–73.9% in the rotigotine group and 50%–60% in the placebo group. Of these reported adverse events, nausea was the most common, followed by headache, nasopharyngitis, application site reaction, somnolence, and dizziness. Nausea was reported in 9.09%–29% of patients receiving rotigotine and 0%–8% of patients receiving placebo. The rates of headache, nasopharyngitis, application site reaction, somnolence, and dizziness were 8%–18%, 8%–18%, 5%–18.8%, 9.09%–10%, and 8%–10%, respectively, in patients receiving rotigotine and 0%–18%, 8%, 0%–4.8%, 9.5%–10%, and 0%–3%, respectively, in patients receiving the placebo. Of these five reviewed studies, two reported no severe adverse events, one did not have data on severe adverse events, and two reported that the rate of severe adverse events was 7.5%–15% in the rotigotine group and 2.5%–10% in the placebo group.

## Discussion

The main results of our meta-analysis are that the PLMI was significantly reduced after therapy with rotigotine in patients with PLMS. Furthermore, rotigotine significantly reduced the PLMI and PLMAI but increased the withdrawal rate in comparison with placebo treatments.

In this study, rotigotine significantly reduced the PLMI and PLMAI when compared with placebo with a difference of 32.105/h in PLMI and 7.160/h in PLMAI, which is similar to the effects of pramipexole and ropinirole. Previous meta-analyses have reported that pramipexole can significantly lower the PLMI by approximately 14.11/h to 30.47/h, and ropinirole can significantly lower the PLMI by approximately 14.11/h to 30.35/h in patients with RLS compared to placebo [36, 37]. One clinical trial showed that pramipexole can lower the PLMI in patients with RBD [60]. Additionally, pramipexole and ropinirole were reported to significantly reduce the PLMAI [29, 61]. Therefore, this study supports the notion that non-ergot dopaminergic receptor agonists may effectively alleviate PLMS, and the efficacy of rotigotine in reducing the PLMI seems to be comparable to that of pramipexole and ropinirole. However, due to the absence of direct head-to-head comparisons, we could not determine which non-ergot dopaminergic receptor agonist is superior to the others with respect to reducing the PLMI and PLMAI.

In this meta-analysis, the difference in means of the PLMI between rotigotine and placebo was −32.105/h in placebo-controlled trial studies, while the difference in means of the PLMI in pre- and post-intervention studies was −5.866/h. The variable effectiveness could be explained by several factors. First, in the pre- and post-intervention studies, PLMS coexisted with different sleep and degenerative disorders, including RLS and RBD in Parkinson’s disease, and the age of the participants ranged from adolescent to elderly [56–58]. Second, in one pre- and post-intervention study, concurrent usage of levodopa was not prohibited because of the patients’ history of Parkinson’s disease[56]. However, it was reported that levodopa may alleviate PLMS, and the concurrent usage of levodopa may mask the effectiveness of rotigotine in reducing PLMI[31]. Third, the duration of titration, duration of maintenance, and total rotigotine dosage were nearly identical in the placebo-controlled trial studies. Conversely, the duration of titration ranged from 3 weeks to 8 weeks, and the duration of maintenance ranged from 2 weeks to 12–20 weeks in the pre- and post-intervention studies [56–58]. In all placebo-controlled trial studies, the maximal dosage of rotigotine was set as 1-3mg/24h, and the mean dosage of rotigotine is 2.31mg, 2.09mg, and 2.47mg, respectively. In contrast, in one pre- and post- intervention study, patients with Parkinson’s disease and RBD, instead of RLS, were enrolled, and a relatively high dosage of rotigotine was used to concomitantly ameliorate their symptoms of Parkinson’s disease with a maximal dosage of 16mg/24h [56], which is completely different from the maximal dosage of 1-3mg/h in other studies [30, 56–59]. The relative heterogeneity of the patients’ background and rotigotine therapy strategies in pre- and post-intervention studies may mask the effectiveness of rotigotine.

Augmentation, manifested by intensified symptoms with earlier onset than baseline, is one of the most common problems in therapy for RLS with dopaminergic receptor agonists [62]. Although augmentation may cause discontinuation of therapy, information regarding augmentation was not available in four studies in which patients with RLS were enrolled. Among non-ergot dopaminergic receptor agonists, the incidence of augmentation is highest in therapy with pramipexole (8.3%–42%) following by rotigotine (2.7%–13%) and ropinirole (2.3%–3.1%) [63–68]. The possible risks for development of augmentation are more severe or more frequent symptoms before therapy, long-term therapy, and usage of immediate-release medication [69, 70]. It was reported that rotigotine might supply a positive effect on augmentation because it can supply a relative stable and continuous plasma level of dopaminergic receptor agonist [71].

Regarding safety considerations, dopaminergic receptor agonists have been reported to significantly increase the withdrawal rate in comparison with placebo, with an OR between 1.37 and 1.82 [36, 37, 72]. Among non-ergot dopaminergic receptor agonists, rotigotine seems to show the highest withdrawal rate, with an OR between 2.5 and 3.08, followed by ropinirole with an OR of 1.76 [37, 72, 73]. Conversely, a non-significant change in withdrawal rate was reported with pramipexole when compared with placebo [36, 37, 73]. This meta-analysis revealed that the OR of the withdrawal rate in patients with rotigotine was 3.421 compared with placebo, which is similar to the findings of other meta-analyses. However, the adverse effects of non-ergot dopaminergic receptor agonists were similar among pramipexole, ropinirole, and rotigotine, including nausea, headache, fatigue, somnolence, vomiting, and dizziness [74, 75]. Although we could not conduct a meta-analysis on the rate of adverse effects due to our limited dataset, the most common adverse effect was nausea, with a variable incidence of 9.09%–29%, which is similar to the previous studies showing possible dosage-related effects [76, 77]. The incidence of nausea in this study is comparable to that seen with pramipexole (15.8%) but lower than with ropinirole (40.3%)[74]. Additionally, application site reactions were reported to be the most common adverse effects in other rotigotine trials, with an incidence of 15%–35% in response to the 1 mg/24 h dosage and 20%–52% under the 3 mg/h dosage [76, 77]. In contrast, the incidence of application site reactions in this study, which ranged from 5%–18.8%, varied between the included studies and was relatively lower than in previous reports.

There were some limitations of this analysis. First, the meta-regression procedure could not be conducted because fewer than five datasets were available in both the primary and secondary outcomes. Second, only 197 participants were enrolled in this meta-analysis, which is a relatively small population. Third, the background heterogeneity, including differences in age, comorbidity, duration of titration and maintenance, and dosage schemes, in these five studies was a potential limitation of the analysis. The heterogeneity of the age of enrolled patients (ranging from adolescents to adults) and the comorbidity (ranging from Parkinson’s disease and RBD to idiopathic and secondary RLS) may affect the generalizability of these findings. All included studies used a duration of rotigotine titration between 3 and 8 weeks and a duration of rotigotine maintenance between 2 and 20 weeks. Therefore, there is no evidence of the long-term efficacy and possible adverse events of rotigotine on the PLMI. Additionally, the dosage-related effects of rotigotine on the PLMI could not be established, because the maximal dosage of rotigotine ranged from 3 mg/24 h (four studies) to 16 mg/24 h (one study). Furthermore, the completely different dosage of rotigotine in patients with Parkinson’s disease and RBD (16mg/24h) may mask the potential effectiveness of rotigotine. Fourth, four of the five included studies were funded by UCB (Monheim am Rhein, Germany), who are the manufacturers of rotigotine. Nevertheless, we consider the results of our meta-analysis to be more convincing than those of the individual studies.

## Conclusions

This meta-analysis revealed the efficacy of rotigotine in alleviating PLMS in terms of the PLMI and PLMAI measurements. However, the high withdrawal rate should be taken into account. Further studies should be conducted to investigate which non-ergot dopaminergic receptor agonist is most effective and tolerable in PLMS patients. Because PLMS may co-occur with other sleep disorders, further studies should be conducted to investigate the efficacy of rotigotine in reducing PLMI in patients with different sleep disorders.

## Supporting information

S1 Fig A: Funnel plot of meta-analysis of the PLMI changes after rotigotine treatment; B: Funnel plot of meta-analysis of the PLMI differences in the rotigotine and placebo-treated groups; C: Funnel plot of meta-analysis of the PLMAI differences in the rotigotine and placebo-treated groups; D: Funnel plot of meta-analysis of withdrawal rate differences in the rotigotine and placebo-treated groups. (PDF)

S1 Table PRISMA checklist of meta-analysis. (DOCX)

S2 Table Excluded studies and rationale. (DOCX)

S3 Table Jadad scores of the included studies. (DOCX)
