# Supplementary figures and images for: The efficacy and tolerability of rotigotine on patients with periodic limb movement in sleep: A systematic review and meta-analysis
Source: PLoS One. 2018 Apr 18;13(4):e0195473. doi: 10.1371/journal.pone.0195473 (PMC5905969; doi:10.1371/journal.pone.0195473)

**Fig S1A**

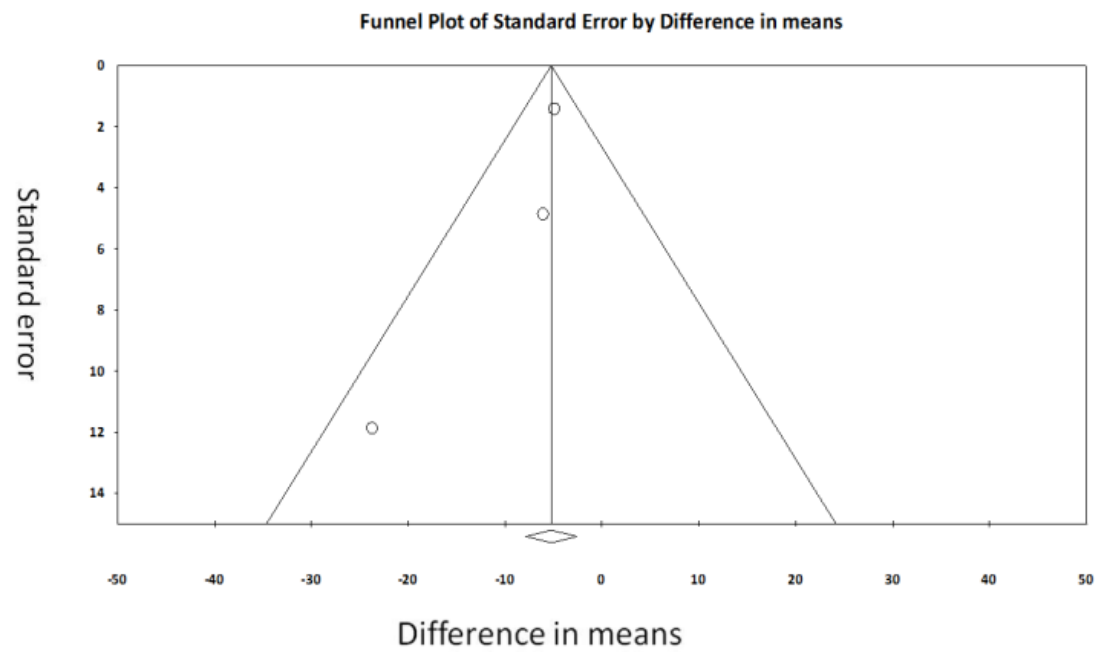

**Fig S1B**

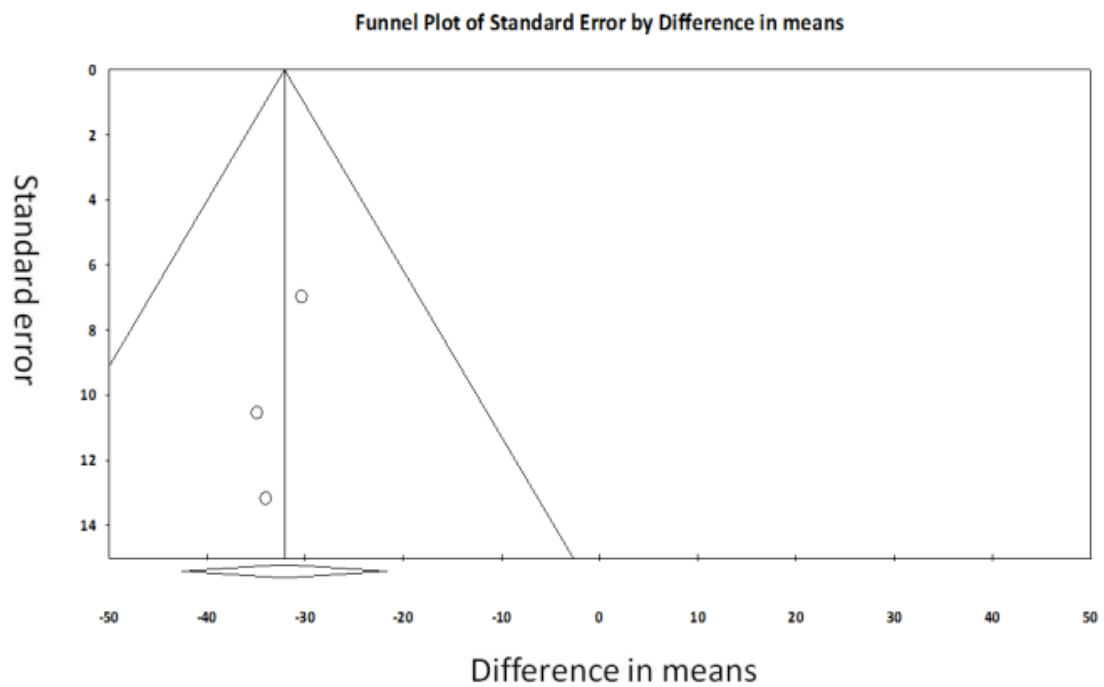

**Fig S1C**

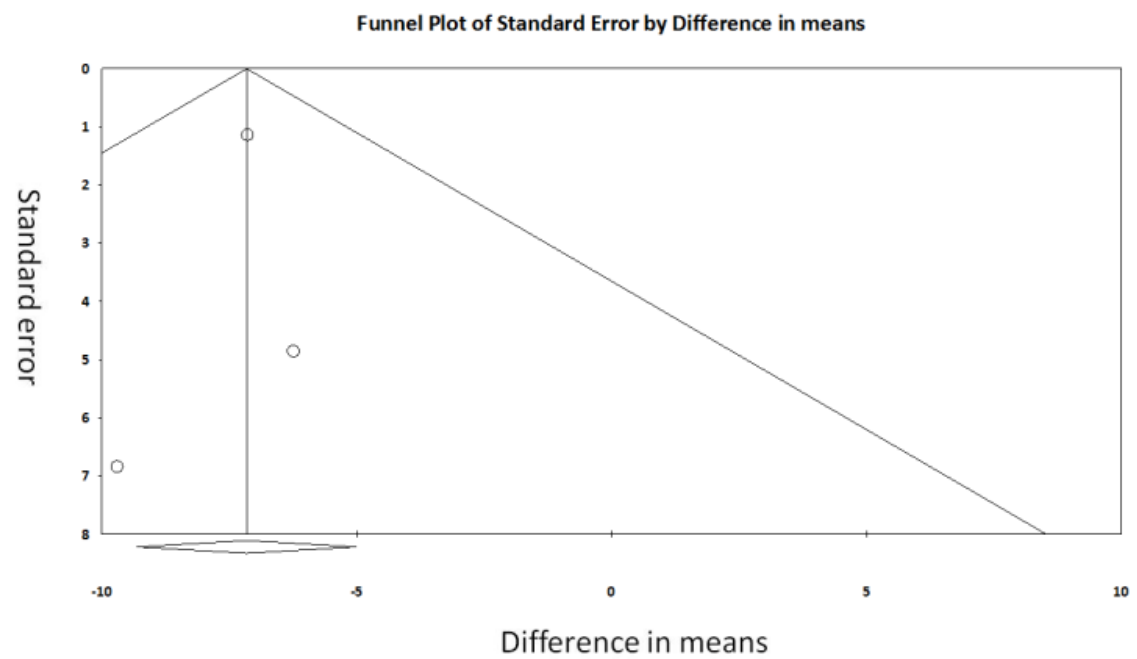

**Fig S1D**

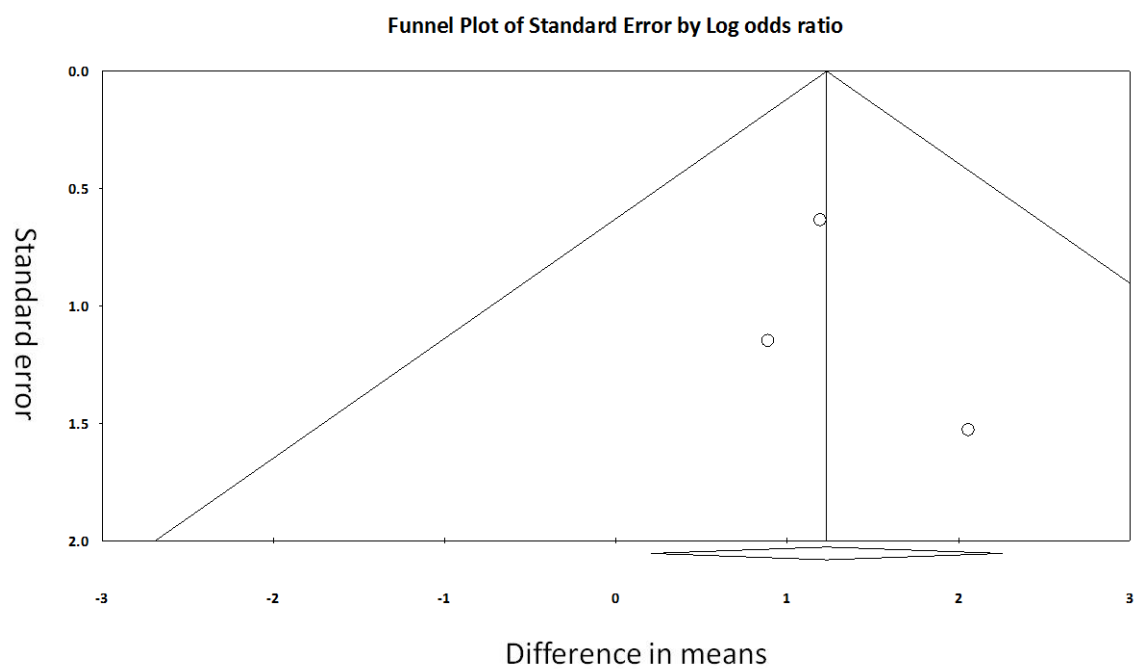

Supplement: S1 Fig — (PDF) [file pone.0195473.s001.pdf]
