# Supplementary material for: The efficacy and tolerability of rotigotine on patients with periodic limb movement in sleep: A systematic review and meta-analysis
Source: PLoS One. 2018 Apr 18;13(4):e0195473. doi: 10.1371/journal.pone.0195473 (PMC5905969; doi:10.1371/journal.pone.0195473)
Supplement: S2 Table — (DOCX) [file pone.0195473.s003.docx]

**S2 Table.** Excluded studies and reasons

Review article (n=7)

Salas RE, Rasquinha R, Gamaldo CE. All the wrong moves: a clinical review of restless legs syndrome, periodic limb movements of sleep and wake, and periodic limb movement disorder. *Clin Chest Med* 2010;31(2):383-95. doi: 10.1016/j.ccm.2010.02.006

Bogan RK. From bench to bedside: An overview of rotigotine for the treatment of restless legs syndrome. *Clin Ther* 2014;36(3):436-55. doi: 10.1016/j.clinthera.2014.01.021

Ferri R, Koo BB, Picchietti DL, et al. Periodic leg movements during sleep: phenotype, neurophysiology, and clinical significance. *Sleep Medicine* 2017

Happe S, Trenkwalder C. Role of dopamine receptor agonists in the treatment of restless legs syndrome. *CNS Drugs* 2004;18(1):27-36.

Silber MH. Sleep-related movement disorders. *Continuum (Minneap Minn)* 2013;19(1 Sleep Disorders):170-84. doi: 10.1212/01.CON.0000427207.13553.68

Fulda S. The Role of Periodic Limb Movements During Sleep in Restless Legs Syndrome: A Selective Update. *Sleep Med Clin* 2015;10(3):241-8, xii. doi: 10.1016/j.jsmc.2015.05.013

Priano L, Gasco MR, Mauro A. Transdermal treatment options for neurological disorders: impact on the elderly. *Drugs Aging* 2006;23(5):357-75.

Not related to PLMS (n=2)

Stiasny-Kolster K, Berg D, Hofmann WE, et al. Effectiveness and tolerability of rotigotine transdermal patch for the treatment of restless legs syndrome in a routine clinical practice setting in Germany. *Sleep Med* 2013;14(6):475-81. doi: 10.1016/j.sleep.2013.02.013

Garcia-Borreguero D, Allen R, Hudson J, et al. Effects of rotigotine on daytime symptoms in patients with primary restless legs syndrome: a randomized, placebo-controlled study. *Curr Med Res Opin* 2016;32(1):77-85. doi: 10.1185/03007995.2015.1103216

Guideline but not clinical trials (n=1)

Vignatelli L, Billiard M, Clarenbach P, et al. EFNS guidelines on management of restless legs syndrome and periodic limb movement disorder in sleep. *Eur J Neurol* 2006;13(10):1049-65. doi: 10.1111/j.1468-1331.2006.01410.x

Case reports (n=1)

Liguori C, Placidi F, Stefani A, et al. Rotigotine effect on sleep in a de novo Parkinson's Disease patient affected by periodic limb movement disorder. *Parkinsonism Relat Disord* 2015;21(12):1476-8. doi: 10.1016/j.parkreldis.2015.10.003

Not related to rotigotine treatment (n=1)

Cassel W, Kesper K, Bauer A, et al. Significant association between systolic and diastolic blood pressure elevations and periodic limb movements in patients with idiopathic restless legs syndrome. *Sleep Med* 2016;17:109-20. doi: 10.1016/j.sleep.2014.12.019

Focus on blood pressure rather than PLMS (n=1)

Grieger F, Schollmayer E, Moran K, et al. The effect of rotigotine on nocturnal blood pressure changes and periodic limb movements of sleep in patients with idiopathic RLS: The encore study. *Sleep Medicine* 2013;14S:e239.
