# Supplementary material for: The efficacy and tolerability of rotigotine on patients with periodic limb movement in sleep: A systematic review and meta-analysis
Source: PLoS One. 2018 Apr 18;13(4):e0195473. doi: 10.1371/journal.pone.0195473 (PMC5905969; doi:10.1371/journal.pone.0195473)
Supplement: S3 Table — (DOCX) [file pone.0195473.s004.docx]

**S3 Table.** Jadad scores of recruited studies

| Author (year) | Randomization | Blindness | Cohort follow up | Total Jadad score | Country |
| --- | --- | --- | --- | --- | --- |
| Wang, Y. (2016) | 0 | 0 | 1 | 1 | China |
| Elshoff, J.P. (2016) | 0 | 0 | 1 | 1 | US |
| Dauvilliers, Y. (2016) | 1 | 1 | 1 | 3 | Multiple countries |
| Oertel, W.H. (2010) | 2 | 2 | 1 | 5 | Multiple countries |
| Bauer, A. (2016) | 1 | 1 | 1 | 3 | Germany |

Abbreviation: US: United States
